# The Role of Callous-Unemotional Traits on Adolescent Positive and Negative Emotional Reactivity: A Longitudinal Community-Based Study

**DOI:** 10.3389/fpsyg.2019.00573

**Published:** 2019-03-15

**Authors:** Erik Truedsson, Christine Fawcett, Victoria Wesevich, Gustaf Gredebäck, Cecilia Wåhlstedt

**Affiliations:** ^1^Department of Psychology, Uppsala University, Uppsala, Sweden; ^2^Department of Obstetrics, Gynecology and Reproductive Sciences, Yale School of Medicine, New Haven, CT, United States

**Keywords:** callous-unemotional traits, emotional reactivity, pupil dilation, longitudinal, community sample

## Abstract

Callous-unemotional (CU) traits are associated with lower emotional reactivity in adolescents. However, since previous studies have focused mainly on reactivity to negative stimuli, it is unclear whether reactivity to positive stimuli is also affected. Further, few studies have addressed the link between CU traits and emotional reactivity in longitudinal community samples, which is important for determining its generalizability and developmental course. In the current study, pupil dilation and self-ratings of arousal and valence were assessed in 100 adolescents (15–17 years) from a community sample, while viewing images with negative and positive valence from the International Affective Pictures System (IAPS). Behavioral traits (CU) were assessed concurrently, as well as at ages 12–15, and 8–9 (subsample, *n* = 68, low levels of prosocial behavior were used as a proxy for CU traits). The results demonstrate that CU traits assessed at ages 12–15 and 8–9 predicted less pupil dilation to both positive and negative images at ages 15–17. Further, CU traits at ages 12–15 and concurrently were associated with less negative valence ratings for negative images and concurrently to less positive valence ratings for positive images. The current findings demonstrate that CU traits are related to lower emotional reactivity to both negative and positive stimuli in adolescents from a community sample.

## Introduction

Children with callous-unemotional (CU) traits are characterized by deficits in empathy and guilt, low distress, low fearfulness, and restricted emotional expression (Frick et al., 2014). CU traits constitute the affective-interpersonal dimension of psychopathy and have been applied downward in age to childhood to identify a developmental precursor to psychopathy (Frick and Ray, 2015). In children and adolescents with conduct problems, elevated CU traits have been concurrently and longitudinally linked to delinquency, aggressive behavior, lower levels of social skills and poorer treatment outcomes (Frick et al., 2014). Further, twin studies indicate that conduct problems in children with elevated CU traits have a considerably stronger genetic influence than in children with normative levels of CU traits (Frick et al., 2014). CU traits are considered to be detectable early in childhood, possibly as early as 2 years of age (Waller et al., 2012) and predict both earlier onset and greater persistence of conduct problems (Dandreaux and Frick, 2009; Rowe et al., 2010b). CU traits have also been shown to be relatively stable throughout childhood and adolescence (*r*s = 0.27–0.84, Frick et al., 2014), although the levels seem to increase in middle adolescence (Essau et al., 2006). Given the negative outcomes associated with elevated levels of CU traits, more knowledge is needed on potential underlying mechanisms/processes related to CU traits.

### Callous-Unemotional Traits and Emotional Reactivity

Several theories on the etiology of psychopathy or CU traits have focused on deficits in reactivity to negatively valenced stimuli (Patrick, 1994; Lykken, 1995; Fowles and Dindo, 2009; Frick and Viding, 2009). That is, CU traits are proposed to be related to lower reactivity to unpleasant stimuli (in particular fear and distress), For instance, Frick and Viding (2009) have proposed that individuals high on CU traits and conduct problems have a specific temperament (lower sensitivity to punishment, fearlessness, lower responsiveness to distress in others) that increases the likelihood of antisocial behavior. These temperamental features hamper the motivation to avoid behaviors that generate harm or distress in others and would therefore explain the tendency for individuals high on CU traits to show little aversion to such behaviors. Such features would further interfere with the normative development of conscience (i.e., guilt and empathy; Frick and Viding, 2009), in line with theoretical accounts of conscience development, in which emotional arousal is viewed as fundamental (Kochanska, 1993; Thompson, 2014).

Yet, it is also possible that lower reactivity to positive stimuli could contribute to behaviors common in those with high CU traits. That is, not being reactive to positive stimuli and situations could impair the establishment of social relationships and a general sense of wellbeing in life that supports connection to social groups. A potential mechanism behind this lower reactivity could be differences in amygdala response. The amygdala is involved in the processing of both positive and negative emotions (Baxter and Murray, 2002) and it has been shown to be less functional in those with higher CU traits (Frick et al., 2014; Herpers et al., 2014). This explanation would also be in line with Blair’s Integrated Emotion Systems model that suggests that psychopathic/callous behaviors are primarily driven by poor functioning of the amygdala and related neural circuitry, which impairs the representations of positive and negative emotional information (Blair, 2004, 2006). More specifically, this results in deficits in approach and avoidance motivation, which then hinders the normal socialization process, driving the development of CU traits.

While much research has examined the link between CU traits and negative emotional reactivity, less has been done to test whether there are also deficits in positive emotional reactivity. That is, research has demonstrated that children and adolescents with elevated levels of CU traits display lower negative emotional reactivity (Frick et al., 2014; Herpers et al., 2014), even on the physiological level (Blair, 1999; Fung et al., 2005; Anastassiou-Hadjicharalambous and Warden, 2008; Marsh et al., 2008; Isen et al., 2010; de Wied et al., 2012; White et al., 2012; Lozier et al., 2014). In studies in which emotional reactivity has been measured with the dot-probe task – a reaction time task that measures how emotional stimuli affects attentional orientation – stimuli of both positive and negative valence have been included and the findings have been that CU traits are specifically related to lower attention/lower reaction time to negatively valanced stimuli (Patrick, 1994; Lykken, 1995; Fowles and Dindo, 2009; Frick and Viding, 2009). However, the dot-probe task is not a direct measure of emotional reactivity; several motoric, affective, and cognitive process are involved in the participants’ reaction times (Vasey et al., 1996). Therefore, associations with CU traits may be different when using a direct measure of emotional reactivity.

There are, to our knowledge, only two studies on physiological emotional reactivity and CU traits which have included stimuli of positive valence (de Wied et al., 2012; Fanti et al., 2016), and the results have been inconsistent. Specifically, Fanti et al. (2016) found that CU traits were associated with lower reactivity to positive stimuli, while de Wied et al. (2012) found no relation. Research on psychopathic traits has also found lower emotional reactivity to positive stimuli in 12- to 18-year-old adolescents (Syngelaki et al., 2013), and in adults (Herpertz et al., 2001). Thus, it is still unclear whether CU traits are associated with lower physiological reactivity to stimuli of positive in addition to negative valence. Evidence from beyond the field of emotional reactivity suggests that this might be the case. For instance, in a meta-analysis, CU traits were related to deficits in emotion recognition across valence (Dawel et al., 2012). In addition, CU traits have been found to be associated with difficulty in perceiving positive social interactions (Fawcett et al., 2016b) as well as with lower responsiveness to parental affection (Dadds et al., 2014) and to reward (Marini and Stickle, 2010).

Self-ratings of emotional reactivity (i.e., arousal and valence) seem to also be affected by CU traits in adolescents. Specifically, in relation to negatively valenced stimuli, the presence of CU traits is related to less negative ratings of valence (Marini and Stickle, 2010; Fanti et al., 2016) and lower ratings of arousal (Sharp et al., 2006; Michonski and Sharp, 2010). For positively valenced visual stimuli, the findings for CU traits have been inconsistent. Masi et al. (2014) found no association, Sharp et al. (2006) found that CU traits were related to increased arousal, and Fanti et al. (2016) found that CU traits were related to less positive ratings of valence. Interestingly, adults with elevated CU traits (measured via the affective dimension of psychopathic traits) respond to emotional stimuli with similar self-ratings of arousal as controls, despite displaying lower physiological reactivity (Patrick et al., 1993; Herpertz et al., 2001). A potential reason for this difference may be that the adults’ responses are more influenced by social desirability than those of adolescents.

### Remaining Questions

The research described in the previous section demonstrates that CU traits are concurrently related to lower emotional reactivity, at least to negative stimuli, in adolescents with conduct problems. Beyond the important question of whether CU traits are associated specifically with lower reactivity to stimuli of negative valence or if reactivity to positive stimuli is also affected, there are several additional areas concerning the relation between CU traits and emotional reactivity that have not yet been thoroughly investigated.

First, to our knowledge, no study has examined whether CU traits in childhood or early adolescence can predict emotional reactivity in late adolescence. From a developmental perspective it is important to acknowledge that age may influence predictive and concurrent associations to behavioral problems as well as potential biological correlates. Therefore, by using longitudinal designs we may gain further knowledge about stability and the predictive value of CU traits in relation to emotional reactivity.

Second, few studies have investigated CU traits dimensionally in relation to emotional reactivity in a community sample. Despite the fact that CU traits vary continuously in community samples (Essau et al., 2006), previous research on CU traits and emotional reactivity has primarily focused on individuals who display extreme scores (e.g., the top 10% of CU traits or forensic/clinical samples). This focus limits the generalizability of the potential effects of CU traits in non-clinical samples, in particular in children and adolescents without elevated levels of Disruptive Behavioral Problems (DBP; see review Herpers et al., 2012). Two notable exceptions have shown that CU traits predict negative behavioral outcomes independently of conduct problems and other psychiatric symptoms in community samples (Dadds et al., 2005; Moran et al., 2008).

### The Current Study

Our aim was to explore whether CU traits are uniquely related to lower emotional reactivity for both positive and negative stimuli, predictively and concurrently in a community sample of adolescents. Emotional reactivity was measured physiologically with pupil dilation as well as with self-ratings of arousal and valence. Pupillometry is an effective way to measure emotional reactivity because pupils dilate with increased allocation of attention or arousal, for example due to emotional intensity or cognitive load (Laeng et al., 2012; Graur and Siegle, 2013). Further, pupillary changes in response to emotional stimuli are considered to be regulated by sympathetic nervous system activity (Bradley et al., 2008) and related to increased amygdala activity (Siegle et al., 2003). The measures have been used in a large range of studies targeting developmental populations (Falck-Ytter, 2008; Chatham et al., 2009; Gredebäck and Melinder, 2010, 2011; Geangu et al., 2011; Gredebäck et al., 2012; Sirois and Jackson, 2012; Hepach and Westermann, 2013; Nyström et al., 2015b; Fawcett et al., 2016a; Hellmer et al., 2016).

We compared adolescents’ emotional reactivity when viewing positive and negative stimuli to their levels of CU traits, both at the current time point and earlier in childhood. In order to ensure that the results were not influenced by co-occurring behavioral symptoms, our analyses for both pupil reactivity and self-ratings of arousal and valence controlled for two symptoms commonly co-occurring with CU traits: DBP and attention deficit hyperactivity disorder (ADHD). We hypothesized that CU traits would be uniquely related to lower physiological emotional reactivity (measured by pupil dilation) for images of both negative and positive valence, based on findings from research with clinical or extreme groups. In contrast, we had no *a priori* hypotheses for the relation between CU traits and self-ratings of arousal and valence, given the inconclusive findings in previous child and adult samples.

## Materials and Methods

### Participants

Participants were initially recruited from two longitudinal community samples (*N* = 867), in which children were drawn either from randomly selected child healthcare centers (Sample A, *n* = 650) or daycare centers (Sample B, *n* = 217) in Sweden at ages 4–6 (Wåhlstedt, 2009; Wåhlstedt and Bohlin, 2010). Given that the high education level and socio-economic status of the participants’ parents in these two original samples (Wåhlstedt, 2009; Wåhlstedt and Bohlin, 2010) would typically lead to very low levels of behavioral problems (including CU traits), subsamples of children with high levels of ADHD symptoms were over-selected from these samples to increase dimensional variability for separate follow up assessments at ages 7–9 (Sample A, *n* = 233; Sample B, *n* = 111). The comorbidity between ADHD and later behavioral problems, which has robust support in the literature, was the justification for this decision (Bélanger et al., 2018). That is, the comorbidity between ADHD and DBP is suggested to be as high as 90% (Dunn and Kronenberger, 2003; Rommelse et al., 2009), and approximately 10–40% of youth with DBP show significant CU traits (Rowe et al., 2010a; Fanti, 2013). At this time point (T_1_), only parent and teacher ratings of children in Sample A were used in the current study. The reason for this was because ratings of the behavioral problems of interest (low levels of prosocial behaviors, hypothesized proxy to tap CU traits; see measure below) were not assessed in Sample B. Five years later, participants from Sample A and Sample B (*n* = 344) were contacted to take part in a shared follow-up (T_2_), which resulted in a sample of 317 children ages 12–15.

A subsample of the participants from the T_2_ follow-up (*N* = 159) was subsequently contacted to take part in an additional follow-up (T_3_). The selection of the subsample was based on aggregated parents and teacher ratings of CU traits from T_2_ to ensure sufficient variability in CU traits. Specifically, high levels of CU traits (see measure below) were defined as scoring in the highest 30% (*n* = 79) and low levels were defined as scoring in the lowest 50% (*n* = 80). Of the 159 parents contacted under these criteria, 70% (*n* = 112); gave permission for their child to participate and complete data was collected for 100 participants (high levels of CU traits *n* = 50 and low levels of CU traits *n* = 50). Reasons for attrition were that the child did not wish to participate (*n* = 4), the child did not show up for assessment (*n* = 2), or we were unable to schedule a time for the child to participate before summer break (*n* = 6).

At T_2_ and T_3_, adolescents’ levels of CU traits (Fanti et al., 2009; Docherty et al., 2016) and ADHD symptoms (DuPaul et al., 1998) were comparable to other large community samples (see Table 1 for descriptive data). This indicates that the oversampling technique used was successful in recruiting a community sample representative of the wider population, despite the fact that the local population from which it was drawn tends to be particularly high on parental education and income level.

**Table 1:** Descriptive data on behavioral ratings.

	*M*	*SD*	Min–max	Range	Cronbach’s alpha
**Behavioral ratings T_1_**	
Parent – SDQ Prosocial behavior T_1_	20.8	3.2	5–25	11–25	0.71
Teacher – SDQ Prosocial behavior T_1_	19.8	3.9	5–25	9–25	0.86
Parent – ODD T_1_	4.0	3.0	0–24	0–14	0.81
Teacher – ODD T_1_	2.2	3.1	0–24	0–14	0.86
Parent – SDQ Conduct problems T_1_	7.6	2.3	5–25	5–13	0.54
Teacher – SDQ Conduct problems T_1_	7.1	2.7	5–25	5–14	0.67
Parent – ADHD T_1_	10.3	8.3	0–54	0–42	0.93
Teacher – ADHD T_1_	7.5	8.3	0–54	0–32	0.93
**Behavioral ratings T_2_**	
Parent – ICU T_2_	18.1	8.6	0–72	3–38	0.86
Teacher – ICU T_2_	22.3	8.7	0–72	2–38	0.86
Parent – ODD T_2_	3.6	3.3	0–24	0–13	0.85
Teacher – ODD T_2_	2.6	3.5	0–24	0–13	0.93
Parent – SDQ Conduct problems T_2_	7.7	2.9	5–25	5–17	0.77
Teacher – SDQ Conduct problems T_2_	7.7	3.2	5–25	5–17	0.82
Parent – ADHD T_2_	8.5	7.7	0–54	0–27	0.93
Teacher – ADHD T_2_	8.7	9.7	0–54	0–39	0.96
**Behavioral ratings T_3_**	
Parent – ICU T_3_	19.8	9.0	0–72	3–42	0.86
Parent – ODD T_3_	3.4	3.4	0–24	0–14	0.88
Parent – SDQ Conduct problems T_3_	7.5	2.8	5–25	5–15	0.76
Parent – ADHD T_3_	7.4	7.4	0–54	0–30	0.93


**n* = 65–98. SDQ, Strengths and Difficulties Questionnaire; ODD, oppositional defiant disorder; ADHD, attention deficit hyperactivity disorder; ICU, Inventory of Callous and Unemotional Traits.*

The final sample of 100 children (45 girls) were 8–9 years at T_1_ (Sample A; *n* = 68, 30 girls, *M* = 8.5 years, *SD* = 2.0 months), 12–15 years at T_2_ (*M* = 13.0 years, *SD* = 6.2 months) and 15–17 years at T_3_ (*M* = 15.8 years, *SD* = 6.5 months). At each time point, parents received detailed written information about the study and returned a signed consent form if they wanted their child to participate. Participants themselves were asked for their assent on the day of testing and were reminded that participation was voluntary and they were free to ask any questions or stop participating at any time without giving a reason. Participants and their parents each received two movie vouchers (worth approximately 20 euros) and teachers received one movie voucher (worth approximately 10 euros) for each assessment in which they participated.

### Materials

#### Questionnaires

Measures regarding CU traits, DBP (i.e., symptoms of ODD and conduct problems) and symptoms of ADHD were collected from parents and teachers at T_1_ and T_2_ and from parents at T_3_. Teacher ratings were not collected at T_3_ since the adolescents no longer had one consistent teacher who knew them well enough to complete the measures. Internal consistency as measured by Cronbach’s alpha ranged from adequate to high for the specific scales (0.71 to 0.96; see Table 1), except for conduct problems at T_1_, which was therefore not included in the analysis. Parent and teacher ratings were correlated at T_1_ (CU traits/low levels of prosocial behavior was used as a proxy, see below, T_1_, *r* = 0.31, *p* = 0.014; ODD T_1_, *r* = 0.32, *p* = 0.012; ADHD T_1_, *r* = 0.28, *p* = 0.030) and T_2_ (CU traits T_2_, *r* = 0.59, *p* < 0.001; DBP T_2_, *r* = 0.23, *p* = 0.035; ADHD T_2_, *r* = 0.41, *p* < 0.001), thus aggregated scores were used at these time points.

##### CU traits

To measure CU traits, the Inventory of Callous-Unemotional Traits (ICU; Frick, 2004) was used at T_2_ and T_3_. The ICU includes 24 items on a 4-point Likert scale ranging from 0 (not at all true) to 3 (definitely true), with higher scores indicating greater CU traits. Previous research has verified the validity of the ICU in community samples of children and adolescents (Roose et al., 2010; Fanti and Centifanti, 2014). At T_1_ a reverse of the subscale prosocial behavior (i.e., low levels of prosocial behavior) from the Strengths and Difficulties Questionnaire (SDQ; Goodman, 1997) was used as a proxy for CU traits since the ICU was not completed. The subscale includes five items, and ratings were made on a 5-point scale ranging from 1 (does not apply at all) to 5 (applies very well). The subscale has been found to be a reliable indicator of CU traits and to load on the same factor as CU measures in factor analyses (Dadds et al., 2005; Pasalich et al., 2011; Hernandez-Lallement et al., 2018).

##### Disruptive behavior problems

To form a broad measure of DBP, scores concerning ODD and conduct problems were standardized and aggregated at T_2_ and T_3_ (parents T_2_, *r* = 0.76; teachers T_2_, *r* = 0.87; parents T_3_, *r* = 0.80, *ps* < 0.001). ODD symptoms were assessed using a well-validated rating scale containing the eight items for ODD as presented in DSM-IV (American Psychiatric Association [APA], 1994). Each item was rated on a 4-point scale ranging from 0 (never or rarely) to 3 (very often). Conduct problems were measured with the conduct problems subscale from the SDQ (Goodman, 1997). The subscale includes five items and ratings were made on a 5-point scale ranging from 1 (does not apply at all) to 5 (applies very well). At T_1,_ only the measure for ODD was used, since the internal consistencies for conduct problems were insufficient for both the parent and teacher ratings (see Table 1). The lack of internal consistency at ages 8–9 for conduct problems is, however, not unexpected considering that the subscale has been found to have low internal consistency at this age (Stone et al., 2010).

##### Attention deficit hyperactivity disorder (ADHD)

Attention deficit hyperactivity disorder symptoms were assessed using a rating scale containing 18 items for ADHD as presented in DSM-IV (American Psychiatric Association [APA], 1994). Each item was rated on a 4-point scale ranging from 0 (never or rarely) to 3 (very often). This measure has been validated and is frequently used in ADHD research (DuPaul et al., 1998).

#### Emotional Reactivity Stimuli

Self-reported ratings of valence and arousal as well as data on pupil dilation (see detailed information below) were collected while the participants viewed emotional stimuli. The stimuli consisted of 38 images from the International Affective Picture System (Lang et al., 2008), which were initially chosen to include 19 pleasant images (for example, a butterfly or children playing) and 19 unpleasant images (for example, weapons, violent behavior; see Supplementary Table S1 for details). The IAPS has been shown to be valid for adolescent samples and provide adequate cross-cultural consistency (Lang and Bradley, 2007).

### Procedure

The IAPS task was presented as one part of a larger battery of 11 tasks measuring various cognitive and social skills. Participants were tested individually in a quiet room at their school. The entire procedure took approximately 1 h and 20 min and was approved by the local ethical board. Gaze was recorded using a Tobii T120 eye tracker with a sampling rate of 120 Hz. Participants sat approximately 50 cm away from the eye tracker. A 9-point calibration was completed before participants viewed the image sequence. Each image filled the screen of the eye tracker and was displayed for 5,000 ms and was followed by the questions regarding valence and arousal presented on the screen. Specifically, participants provided self-ratings of valence (i.e., “how happy or sad does the photo make you”) and arousal (i.e., “how excited or calm does the photo make you”) on a single item Likert scale, ranging from 1 to 9 by selecting a number key (1–9) on a keyboard that was placed approximately 30 cm in front of the participant. For valence, low scores indicated an unpleasant emotional reaction while high scores a pleasant reaction. Concerning arousal, low scores indicated low levels of arousal, and high scores high levels of arousal. The next image was presented directly after the participant had rated both valence and arousal. The 38 images from the IAPS were shown inter-mixed with 12 additional images of angry, fearful, and neutral male and female faces which are not included in the current study. The 50 images were displayed to each participant in one of four possible semi-randomized orders. At T_1_ and T_2_, parent and teacher questionnaires were completed by regular post, and at T_3_ parent questionnaires were completed online.

### Data Management and Reduction

Prior to analysis, questionnaire data were screened for outliers, defined as values ±3 *SD* and replaced with the value that was the next most extreme, in line with the Winsorizing procedure (Chen et al., 2001). Eye tracking data was processed in the MATLAB-based open source program TimeStudio version 3.15^1^ (Nyström et al., 2015a; the processing procedure used in the current study can be downloaded into TimeStudio using uwid: ts-fd3-dd9). First, gaps in the data of up to 10 samples (83 ms) were interpolated linearly and the data was smoothed using a moving median over six samples (50 ms) and then a moving average over six samples (50 ms). Then the values for each trial were adjusted using a baseline of the first 500 ms of the image display, which was subtracted from the average pupil size from the analysis period of 1 to 3 s. The baseline helps to account for differences in light across individual images. Trials with less than 50% of pupil size data recorded were excluded.

### Statistical Analyses

All relations between predictors and outcome were analyzed per trial using linear mixed-effects models in R (version 3.4.3, R; R Development Core Team, 2014) with the package lme4 (version 1.1-15; Bates et al., 2014). All models included a random effect of participant. Random effects are beneficial for taking into account the individual variability of participants or trials to strengthen analyses (Baayen et al., 2008). In the main analyses, behavioral ratings from parents and teachers at T_1_ and T_2_, and from parents at T_3_ were used. Data are available from the authors upon reasonable request.

## Results

Descriptive statistics for behavioral symptoms, pupil data and control variables are presented in Table 1. Correlations for behavioral symptoms ratings used in the analyses are shown in Table 2.

**Table 2:** Inter-correlations between aggregated behavioral ratings.

	1	2	3	4	5	6	7	8	9
1. SDQ Prosocial behavior (reversed) $T_{1}^{a}$	_	0.38^∗∗^	0.11	0.42^∗∗∗^	0.47^∗∗∗^	0.20	0.24	0.37^∗∗^	0.19
2. ODD $T_{1}^{a}$		–	0.53^∗∗∗^	0.32^∗∗^	0.49^∗∗∗^	0.31^∗∗^	0.25^∗^	0.40^∗∗∗^	0.25^∗^
3. ADHD $T_{1}^{a}$			–	0.35^∗∗∗^	0.36^∗∗∗^	0.53^∗∗∗^	0.41^∗∗∗^	0.38^∗∗∗^	0.54^∗∗∗^
4. ICU $T_{2}^{a}$				–	0.68^∗∗∗^	0.59^∗∗∗^	0.64^∗∗∗^	0.58^∗∗∗^	0.42^∗∗∗^
5. DBP $T_{2}^{a}$					–	0.72^∗∗∗^	0.50^∗∗∗^	0.76^∗∗∗^	0.54^∗∗∗^
6. ADHD $T_{2}^{a}$						–	0.49^∗∗∗^	0.58^∗∗∗^	0.42^∗∗∗^
7. ICU $T_{3}^{b}$							–	0.69^∗∗∗^	0.63^∗∗∗^
8. DBP $T_{3}^{b}$								–	0.72^∗∗∗^
9. ADHD $T_{3}^{b}$									–


**n* = 65–98. ^∗^*p* < 0.05, ^∗∗^*p* < 0.01, ^∗∗∗^*p* < 0.001. SDQ, Strengths and Difficulties Questionnaire; ODD, oppositional defiant disorder; ADHD, attention deficit hyperactivity disorder; ICU, Inventory of Callous and Unemotional Traits. ^*a*^Aggregated parent and teacher ratings, ^*b*^parent ratings.*

### Preliminary Analyses

**FIGURE 1:**
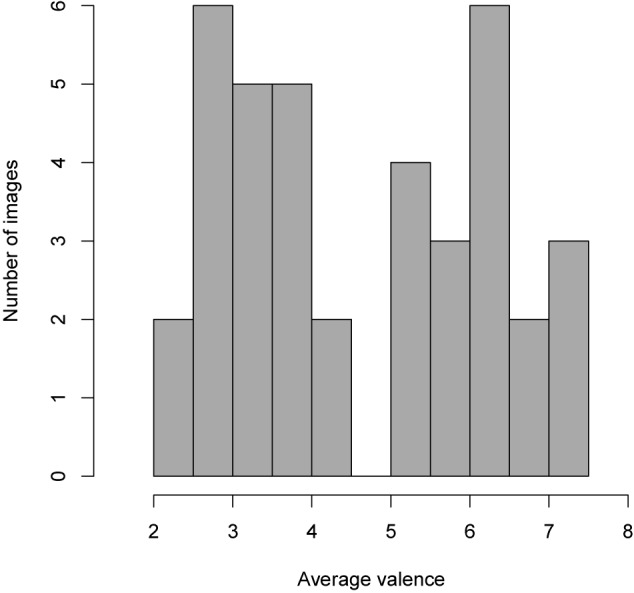
Histogram showing the distribution of mean self-ratings of valence for the 38 images from the IAPS used in the present study. Visual inspection led to dividing the images into high and low valence groups with a split at the rating 5.

We first compared the valence and arousal ratings of the participants in the current study with norm ratings for adults from the IAPS manual (Lang et al., 2008). There was significant agreement between IAPS norms and the average ratings from our participants for both arousal [*ICC*(37) = 0.74, *p* < 0.001] and valence [*ICC*(37) = 0.94, *p* < 0.001], as measured using Intraclass Correlations. Before proceeding to the main analysis, the images from the IAPS were spilt into two groups based on visual inspection of the histogram of self-ratings of valence which clearly shows a bimodal distribution (low, *n* = 20, high, *n* = 18; see Figure 1). We also examined the internal consistency by calculating Cronbach’s coefficient alpha for the ratings of the two groups of images (low and high valence). The coefficient alphas for low valence images were 0.89 for arousal and 0.92 for valence; for high valence images, they were 0.74 for arousal and 0.75 for valence (*p*s < 0.001).

The IAPS images were assessed for average image luminance as well as contrast at high- and low-spatial frequency (HSF and LSF, respectively) to examine whether they could be confounded with arousal and valence (Delplanque et al., 2007). Luminance, HSF contrast, and LSF contrast were all found to be related to ratings of both arousal (luminance: *b* = -0.011, *SE* = 0.001, 95% CI [-0.013, -0.010], *t* = -14.21, *p* < 0.001; HSF contrast: *b* = 3.855, *SE* = 0.379, 95% CI [3.113, 4.597], *t* = 10.18, *p* < 0.001; LSF contrast: *b* = 5.980, *SE* = 0.562, 95% CI [4.879, 7.081], *t* = 10.65, *p* < 0.001) and valence (luminance: *b* = 0.017, *SE* = 0.001, 95% CI [0.016, 0.019], *t* = 19.15, *p* < 0.001; HSF contrast: *b* = -7.028, *SE* = 0.455, 95% CI [-7.919, -6.137], *t* = -15.46, *p* < 0.001; LSF contrast: *b* = -7.996, *SE* = 0.685, 95% CI [-9.338, -6.653], *t* = -11.68, *p* < 0.001). Thus, all three were included as control variables in the main analyses.

Sex of participant was not related to pupil dilation in a model including sex as predictor and pupil dilation as outcome (*b* = -0.001, *SE* = 0.023, 95% CI [-0.047, 0.044], *t* = -0.052, *p* > 0.1), or to self-ratings of valence in a model including sex as predictor and self-ratings of valence as outcome (*b* = -0.060, *SE* = 0.085, 95% CI [-0.226, 0.105], *t* = -0.712, *p* > 0.1), therefore sex was not included in further analyses with pupil dilation or valence as outcome. There was, however, a marginally significant effect of sex of participant on self-ratings of arousal in a model including sex as predictor and self-ratings of arousal as outcome (*b* = 0.232, *SE* = 0.123, 95% CI [-0.009, 0.472], *t* = 1.887, *p* = 0.062) with females (*M* = 5.45, *SD* = 1.90) reporting higher levels of arousal than males (*M* = 5.22, *SD* = 1.89). Sex was therefore included as a control in the analyses with self-ratings of arousal as outcome.

**FIGURE 2:**
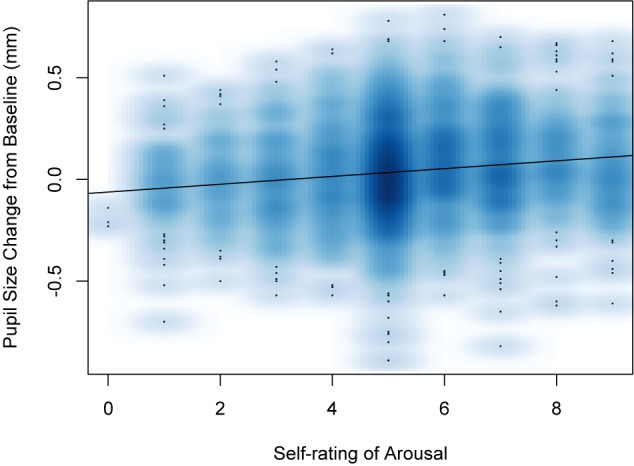
Smoothed scatterplot showing the relationship between participants’ self-ratings of arousal and their change in pupil size from baseline on each trial. The regression indicates a significant relation between the two measures.

To confirm that arousal as measured by self-ratings was related to arousal as measured by pupil dilation, a model in which self-rating of arousal was the predictor and pupil dilation was the outcome was tested. Self-ratings of arousal did significantly predict pupil dilation (*b* = 0.019, *SE* = 0.002, 95% CI [0.015, 0.023], *t* = 9.48, *p* < 0.001; see Figure 2).

### Predictive and Concurrent Relations Between Behavioral Symptoms and Pupil Dilation to Positively and Negatively Valenced Images

**FIGURE 3:**
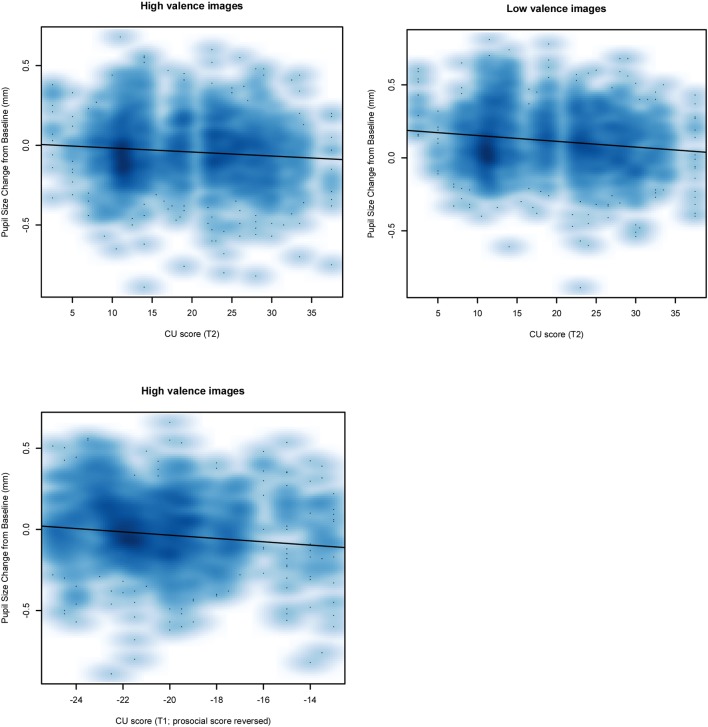
Smoothed scatterplots showing the significant relationship between participants’ CU scores and their change in pupil size from baseline on each trial.

Linear mixed models including the behavioral symptoms of CU traits, DBP, and ADHD as predictors, and pupil dilation as outcome were used to investigate emotional reactivity predictively (T_2_) and concurrently (T_3_). Predictively, CU traits (T_2_) were related to less pupil dilation relative to baseline for images of both negative (*b* = -0.005, *SE* = 0.002, 95% CI [-0.010, -0.001], *t* = -2.253, *p* = 0.027) and positive (*b* = -0.004, *SE* = 0.002, 95% CI [-0.008, -0.0003], *t* = -2.090, *p* = 0.040) valence (see Figure 3). Concurrently (T_3_), none of the behavioral symptoms were related to pupil dilation (*p’*s > 0.1).

For the subsample (*n* = 68) for which low prosocial behaviors (i.e., reversing the prosocial behavior scale as a proxy for CU traits) were assessed at T_1_, a linear mixed model including low prosocial behavior, ODD, and ADHD as predictors, and pupil dilation as outcome was tested. Low prosocial behavior was related to less pupil dilation relative to baseline for images of positive valence (*b* = -0.012, *SE* = 0.005, 95% CI [-0.023, -0.002], *t* = -2.370, *p* = 0.021, see Figure 3), but not for images of negative valence (*p* > 0.1).

### Predictive and Concurrent Relations Between Behavioral Symptoms and Self-Ratings of Arousal and Valence to Positively and Negatively Valenced Images

#### Arousal

Linear mixed models including CU traits, DBP, ADHD as predictors, and self-ratings of arousal as outcome were conducted predictively (T_2_) and concurrently (T_3_). For negatively valenced images, CU traits were predictively (*b* = -0.030, *SE* = 0.015, 95% CI [-0.059, 0.000], *t* = -1.928, *p* = 0.057) and concurrently (*b* = -0.024, *SE* = 0.014, 95% CI [-0.052, 0.004], *t* = -1.668, *p* = 0.099) related to lower ratings of arousal with marginal significance. For positively valenced images, there were no significant relations between self-ratings of arousal and CU traits, however, there were significant associations for several control variables. Predictively, ADHD symptoms were uniquely related to higher ratings of arousal (*b* = 0.052, *SE* = 0.018, 95% CI [0.019, 0.086], *t* = 2.988, *p* = 0.004), as was participant sex (*b* = 0.369, *SE* = 0.177, 95% CI [0.028, 0.710], *t* = 2.088, *p* = 0.040), with females rating higher arousal levels than males. Concurrently, DBP were significantly related to lower ratings of arousal (*b* = -0.327, *SE* = 0.154, 95% CI [-0.624, -0.029], *t* = -2.116, *p* = 0.037) and ADHD was marginally related to higher ratings of arousal (*b* = 0.035, *SE* = 0.018, 95% CI [0.001, 0.070], *t* = 1.972, *p* = 0.052). Finally, for the subsample for which low prosocial behaviors (i.e., proxy for CU traits) were assessed at T_1_, a linear mixed model including prosocial behavior, ODD, ADHD as predictors, and self-ratings of arousal as outcome was tested. No relations were found (*p*s > 0.1).

#### Valence

Linear mixed models including CU traits, DBP, ADHD as predictors, and self-ratings of valence as outcome were conducted predictively (T_2_) and concurrently (T_3_). For negatively valenced images, CU traits were predictively (*b* = 0.033, *SE* = 0.015, 95% CI [0.003, 0.063], *t* = 2.203, *p* = 0.030) and concurrently (*b* = 0.032, *SE* = 0.015, 95% CI [-0.003, 0.061], *t* = 2.195, *p* = 0.031) related to less negative ratings of valence. In addition, ADHD symptoms were predictively related to less negative ratings of valence with marginal significance (*b* = 0.033, *SE* = 0.017, 95% CI [-0.001, 0.068], *t* = 1.932, *p* = 0.056). For positively valenced images, CU traits were concurrently (*b* = -0.026, *SE* = 0.010, 95% CI [-0.046, -0.006], *t* = -2.585, *p* = 0.011) related to less positive ratings of valence. No relations were found predictively (*p*’s > 0.1).

In addition, for the subsample for which low prosocial behaviors (i.e., proxy for CU traits) were assessed at T_1_, linear mixed models including prosocial behavior, ODD, ADHD as predictors, and self-ratings of valence as outcome were tested. No relations were found (*p*’s > 0.1).

## Discussion

The current study examined the relation between positive and negative emotional reactivity and CU traits in adolescents from a community sample, both predictively and concurrently, while controlling for DBP and ADHD symptoms. In line with expectations, we found that CU traits were predictively associated with lower emotional reactivity (less pupil dilation) when viewing both positive and negative images from the IAPS. Specifically, lower emotional reactivity to images of both positive and negative valence at ages 15–17 was associated with CU traits at ages 12–15. In addition, low levels of prosocial behavior (proxy for CU traits) at ages 8–9 were associated with lower emotional reactivity to images of positive valence at ages 15–17. Interestingly, no associations were found between CU traits and emotional reactivity concurrently.

Given the previously mixed findings on CU traits and self-ratings of arousal, we did not have a clear hypothesis regarding potential associations for this part of our study. The results showed that CU traits were marginally associated with lower ratings of arousal to negative images, predictively, at ages 12–15, and concurrently in line with previous findings (Sharp et al., 2006; Michonski and Sharp, 2010; de Wied et al., 2012). Further, ADHD symptoms at ages 12–15 and concurrently were related to higher ratings of arousal to positive images, which is consistent with a recent study finding an association between increased positive emotionality (rated by parents) and ADHD symptoms (Forslund et al., 2016). In addition, DBP were concurrently related to lower ratings of arousal to positive images. Together these findings suggest that self-ratings of arousal have some relation to CU traits, but that the physiological measure of emotional reactivity, pupil dilation, is a more direct way to assess arousal and is less likely to be influenced by other participant factors, making the relation between arousal and CU traits more clear.

The current findings on emotional reactivity fail to provide support for the prevalent view that high levels of CU (or psychopathic) traits are specifically related to lower reactivity to stimuli of negative valence (Frick et al., 2014; Herpers et al., 2014) and instead suggest that emotional reactivity to positive images is also affected by CU traits. Only two prior studies on emotional reactivity and CU traits have included stimuli with positive valence (de Wied et al., 2012; Fanti et al., 2016) and the results have been mixed. Specifically, Fanti et al. (2016) found that CU traits were associated with lower neurophysiological activity in response to both positive and negative stimuli: videos depicting both violent and comedy scenes. Whereas de Wied et al. (2012) did not find conclusive evidence for a relation between CU and emotional reactivity to happiness. However, the latter study had some limitations, which constrain the ability to draw clear conclusions. First, the statistical power was limited since the analyses were conducted between groups with low sample sizes. Second, unique variance in relation to CU traits may have been masked by not taking into account the shared variance between DBP and CU traits (i.e., not controlling for DBP when examining the relation between CU traits and emotional reactivity). Thus our results provide a more conclusive statement on the relation between CU traits and positive emotional reactivity, adding to findings that have found similar relations between positive emotional reactivity and psychopathic traits in adolescents, ages 12–18 (Syngelaki et al., 2013), and in adults (Herpertz et al., 2001). Further, the findings are in line with research concerning others areas of socio-emotional processing in which CU traits have been found to be associated with lower ability to process positive affect, such as difficulty in perceiving positive social interactions (Fawcett et al., 2016b) and lower responsiveness to parental affection (Dadds et al., 2014).

To further advance developmental theories on CU traits, it may be important to consider the potential role that deficits in both perceiving and experiencing positive affect may have. Thus, while previous findings have mainly demonstrated that deficits in emotional reactivity are specific to stimuli of negative valence (in particular fear and distress) our current findings suggest that CU traits may be related to lower reactivity across valence, consistent with the Integrated Emotion Systems model proposed by Blair (2004, 2006). Impaired representation of emotional information of both positive and negative affect might contribute to CU traits, for example in that decreased approach and avoidance motivation (seeking situations associated with happiness and avoid those associated with fear and distress) might hamper normal socialization and social interaction.

Contrary to our expectations, CU traits were not concurrently related to lower emotional reactivity measured by pupil dilation. This is inconsistent with prior research with physiological measures of emotional reactivity, which have found CU traits related to lower reactivity in adolescence (Blair, 1999; Fung et al., 2005; Marsh et al., 2008; Isen et al., 2010; de Wied et al., 2012; White et al., 2012; Lozier et al., 2014). However, these studies have included clinical or extreme groups, which may have increased the potential to find relations between CU traits and emotional reactivity. It is possible that the discrepant findings between predictive and concurrent ratings of CU traits and physiological emotional reactivity in the current study are influenced by developmental differences (pre/early-adolescence vs. middle adolescence). That is, the normative levels of CU traits in childhood have been suggested to vary depending on age (Edens et al., 2001; Seagrave and Grisso, 2002), which is consistent with a community sample study (*n* = 1,433), in which adolescents 15–16 years old had significantly higher CU scores than adolescents either 13–14 or 17–18 years old (Essau et al., 2006). It is therefore possible that measures of CU traits rated during middle adolescence may to some degree capture behaviors that are specific to the age group, rather than underlying CU traits. Even the emotional reactivity of children changes during development. For example, brain imaging studies have shown that emotional reactivity decreases with age, which means that aversive affective cues are instead interpreted in a more specialized and cognitive manner when children become older (Michalska and Davis, 2018). However, there may also be other factors that underlie the discrepancy. For instance, the validity of parent and teacher reports tend to decrease from childhood to adolescence (Kamphaus and Frick, 1996), suggesting that ratings are less reliable overall in adolescence. Further, it is possible that the mechanism which results in the decreased automatic emotional reactivity is one that develops over time, meaning that the more relevant levels of CU traits are actually those from earlier in development, not those from the current timepoint.

The current study has several strengths in relation to previous work in this area. One strength is that we examined the role of CU traits in relation to emotional reactivity in a community sample. Prior research examining CU traits has mainly concerned children and adolescents that have already been identified as having problematic behavior (e.g., Pardini et al., 2003; Stickle et al., 2009). Thus, by using community sample, we are able to show that CU traits are related to lower emotional reactivity even in a non-clinical population. However, the use of a community sample may in turn put constraints on the ability to generalize the findings to clinical populations.

A second strength is that we examined relations between emotional reactivity and CU traits developmentally over late childhood and into adolescence. This study is the first to our knowledge that explores predictive relations between CU traits and emotional reactivity. However, given that we only measured emotional reactivity at the latest time point, ages 15–17, we cannot, for instance, provide insight into concurrent relations between CU traits and emotional reactivity earlier in childhood or shed light on how emotional reactivity in childhood is associated to CU traits in adolescence. Further, we must take into account that the earliest time point used low levels of prosocial behavior for a proxy of CU traits and it might not fully tap into all the aspects of CU traits, in particular the unemotional dimension. Nevertheless, the subscale has been found to be a reliable indicator of CU traits (Dadds et al., 2005; Pasalich et al., 2011) and been used as a proxy of CU traits (Musser et al., 2013). These are all important topics to be addressed in future research.

Finally, our study controlled for common comorbid symptoms in children and adolescents with CU traits, such as ADHD and DBP. This is important because it is possible for DBP to have suppressing effects on CU traits (Viding et al., 2012; Lozier et al., 2014) which can mask relations between CU traits and other factors. In addition, it is valuable to acquire knowledge of the cognitive and emotional processes that are uniquely related to CU traits. One reason for this is that children and adolescents with DBP and high levels of CU traits, compared with DBP and normative levels of CU traits, show poor response to treatments (Frick et al., 2014; Frick, 2016; Pisano et al., 2017). By controlling for common comorbid symptoms, we may contribute to better treatments for individuals with high levels of CU traits.

Despite our efforts to control for the low-level perceptual features of the images (i.e., contrast and luminance), it is still possible that the measures we used are not completely accurate due to differences in scanning patterns. That is, if different participants scan the images differently, they may spend more time in areas that are higher or lower on these features than for the images as a whole. We believe this is unlikely to affect our results for several reasons. First, pupil size correlated well with self-ratings of arousal, suggesting that measured pupil dilation was related to participants’ subjective emotional reactivity. Second, there was significant variability across the images such that there was no consistent difference for arousing aspects of the image to be lighter or darker than the background. Thus, even if individuals scanned the images differently, it would be unlikely to result in confounded pupil size over trials.

A second possible limitation is that we cannot reliably test the effects of having social content in the stimulus images. The images were not initially selected to be able to test this factor and thus were not evenly distributed as far as valence and arousal. In addition, there were 12 images of emotional and neutral faces inter-mixed with the images used in the current study. Viewing these faces could potentially have increased the social nature of the task as a whole. Future research could potentially examine whether the effect of CU traits on emotional responses vary depending on the social content and context of the stimuli.

## Conclusion

The current study extends previous findings on emotional reactivity in relation to CU traits. First, we show that CU traits are dimensionally related to lower emotional reactivity in adolescents from a community sample, for both positive and negative stimuli, providing support for the Integrated Emotion Systems model (Blair, 2004, 2006). That is, a lack of engagement and reaction to positive situations, not only negative ones, likely contribute to CU traits and their associated outcomes. Second, we demonstrate that the relation between CU traits and emotional reactivity changes over development. Together these findings may be important for improving treatments for children and adolescents with elevated CU traits.

## Data Availability

The datasets generated for this study are available on request to the corresponding author.

## Ethics Statement

This study has been designed in accordance with the ethics rules of the Swedish Research Council, and reviewed and approved by the Regional Ethics Committee in Uppsala, Sweden (ref. 2010/190).

## Author Contributions

All authors contributed to the study design. ET, CW, and VW collected the data. ET and CF performed the data analysis and ET drafted the manuscript. CF, CW, GG, and VW provided critical revisions. All authors approved the final version of the manuscript for submission.

## Conflict of Interest Statement

The authors declare that the research was conducted in the absence of any commercial or financial relationships that could be construed as a potential conflict of interest.

## Footnotes

1 timestudioproject.com
