# Supplementary material for: The Role of Callous-Unemotional Traits on Adolescent Positive and Negative Emotional Reactivity: A Longitudinal Community-Based Study
Source: Front Psychol. 2019 Mar 15;10:573. doi: 10.3389/fpsyg.2019.00573 (PMC6428776; doi:10.3389/fpsyg.2019.00573)
Supplement: Supplementary file 1 [file Data_Sheet_1.docx]

Supplementary Information

Table S1. Descriptive information for IAPS images used in the study

| Image number | Content | Valence group | Valence rating (M) | Arousal rating (M) |
| --- | --- | --- | --- | --- |
| 1340 | women | high | 7.21 | 4.75 |
| 1440 | seal | high | 7.43 | 4.61 |
| 1525 | dog barking | low | 3.80 | 6.61 |
| 1602 | butterfly | high | 6.38 | 3.43 |
| 1810 | hippo | high | 6.41 | 4.45 |
| 2205 | elders & hospital bed | low | 2.58 | 4.53 |
| 2387 | children | high | 6.21 | 3.97 |
| 2521 | man with dog | high | 5.20 | 4.10 |
| 2530 | man & woman biking | high | 7.17 | 3.99 |
| 2682 | person with baton | low | 4.31 | 4.48 |
| 2683 | armed riot | low | 3.13 | 6.21 |
| 2691 | man throwing rock in riot | low | 3.49 | 5.85 |
| 2694 | person under arrest | low | 3.91 | 5.05 |
| 2700 | people in distress | low | 3.48 | 4.77 |
| 2703 | children in distress | low | 2.15 | 5.78 |
| 2900.1 | child upset | low | 2.97 | 5.09 |
| 5814 | beach & palmtrees | high | 5.60 | 4.82 |
| 5831 | adult & child looking at birds | high | 6.81 | 4.43 |
| 5875 | man with bike | high | 5.88 | 3.29 |
| 6212 | man with gun & boy running | low | 2.31 | 6.01 |
| 6230 | hand pointing gun | low | 3.60 | 7.35 |
| 6231 | masked person pointing gun | low | 3.58 | 6.82 |
| 6315 | man attacking woman | low | 2.87 | 6.38 |
| 7004 | spoon | high | 5.50 | 2.00 |
| 7009 | mug | high | 5.24 | 3.01 |
| 7032 | shoes | high | 5.17 | 3.81 |
| 7440 | person using bbq | high | 6.57 | 4.70 |
| 7489 | boat and city | high | 6.20 | 4.49 |
| 7509 | hand painting | high | 5.75 | 3.43 |
| 8460 | man running with dog | high | 6.14 | 4.55 |
| 8500 | gold | high | 6.13 | 5.60 |
| 9041 | distressed girl in fetal position | low | 2.97 | 4.64 |
| 9220 | man & woman in graveyard | low | 3.06 | 4.00 |
| 9395 | dirty dishes | low | 4.02 | 4.22 |
| 9421 | wounded soldiers | low | 2.86 | 5.04 |
| 9426 | people with bags over their heads | low | 3.12 | 5.28 |
| 9600 | sinking ship | low | 2.99 | 6.46 |
| 9810 | KKK & burning cross | low | 3.56 | 6.62 |
